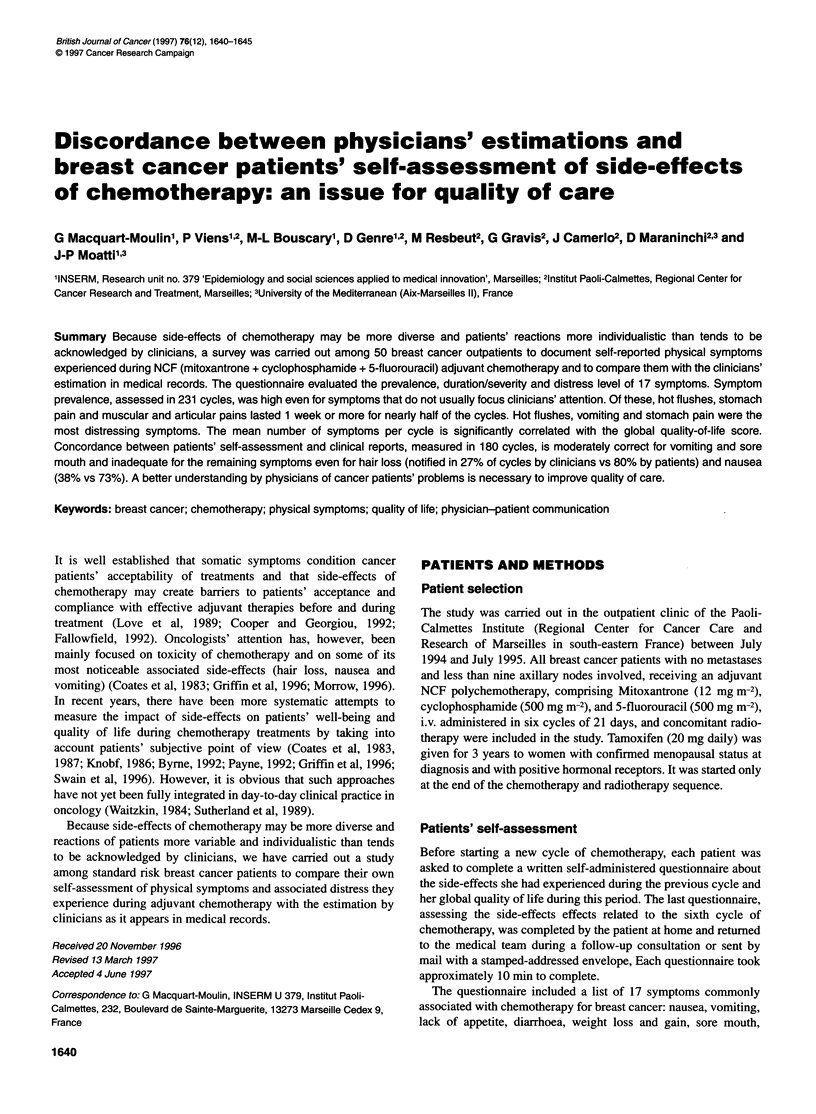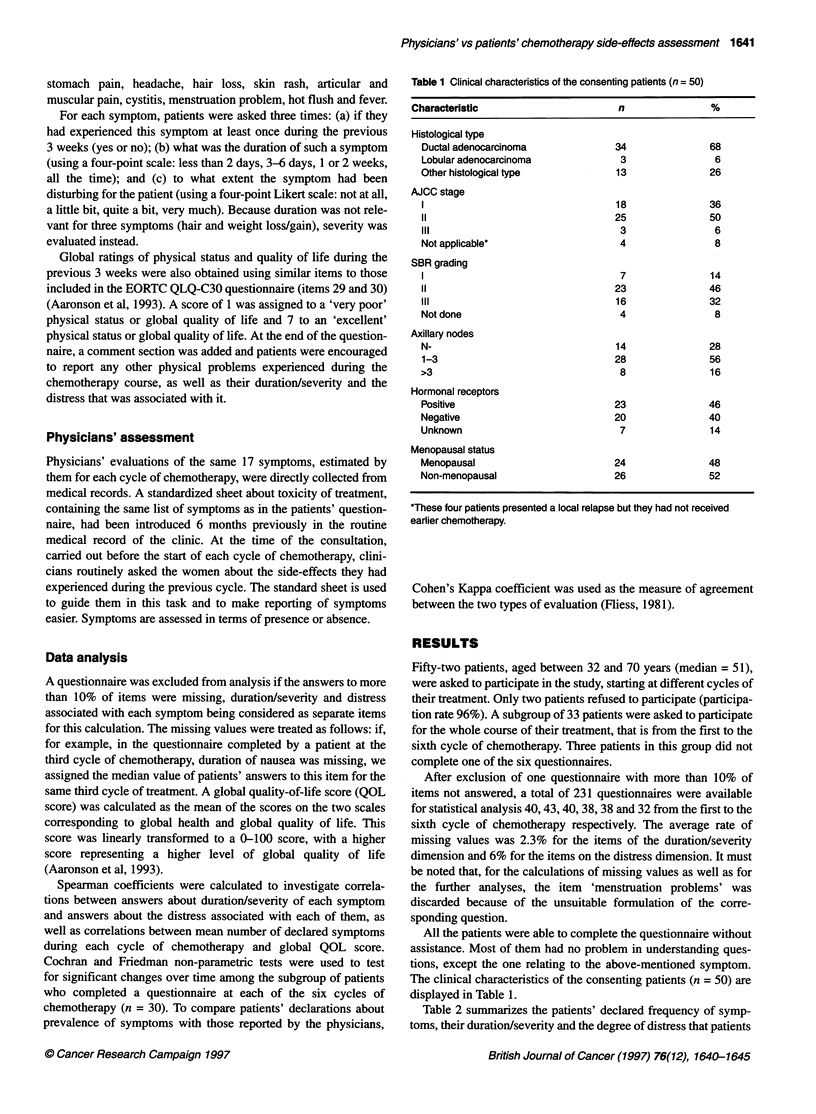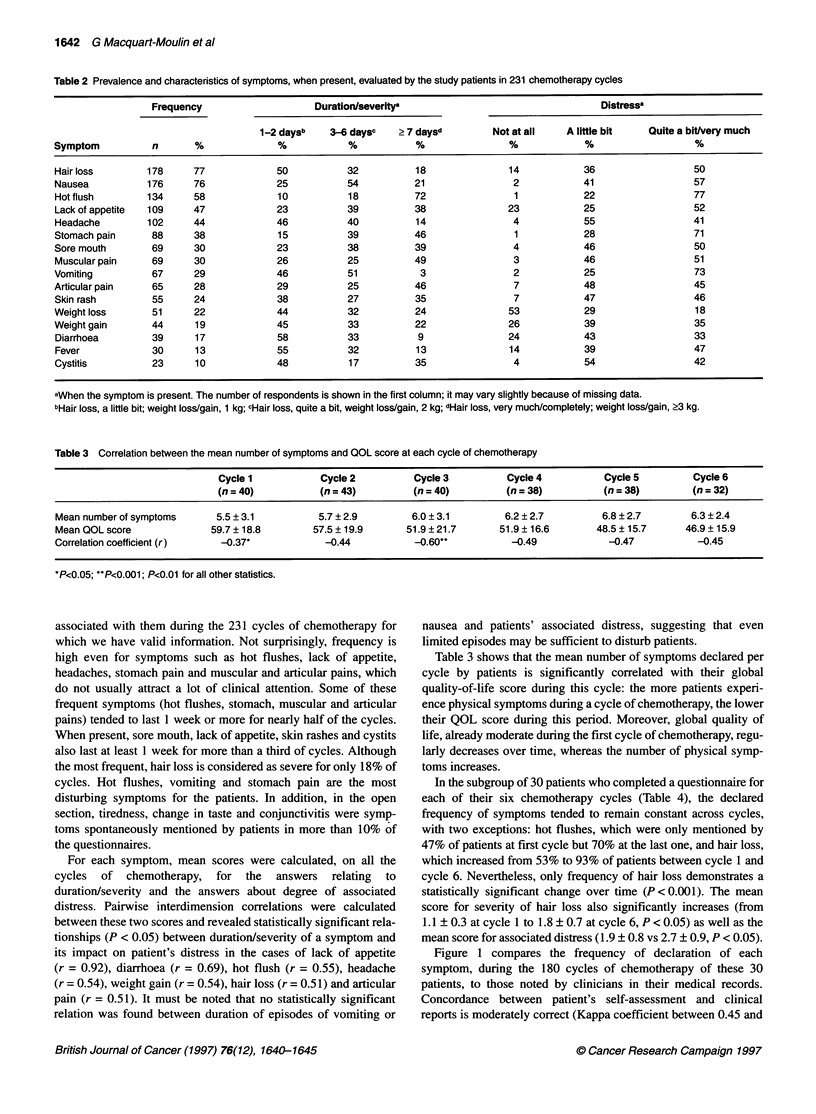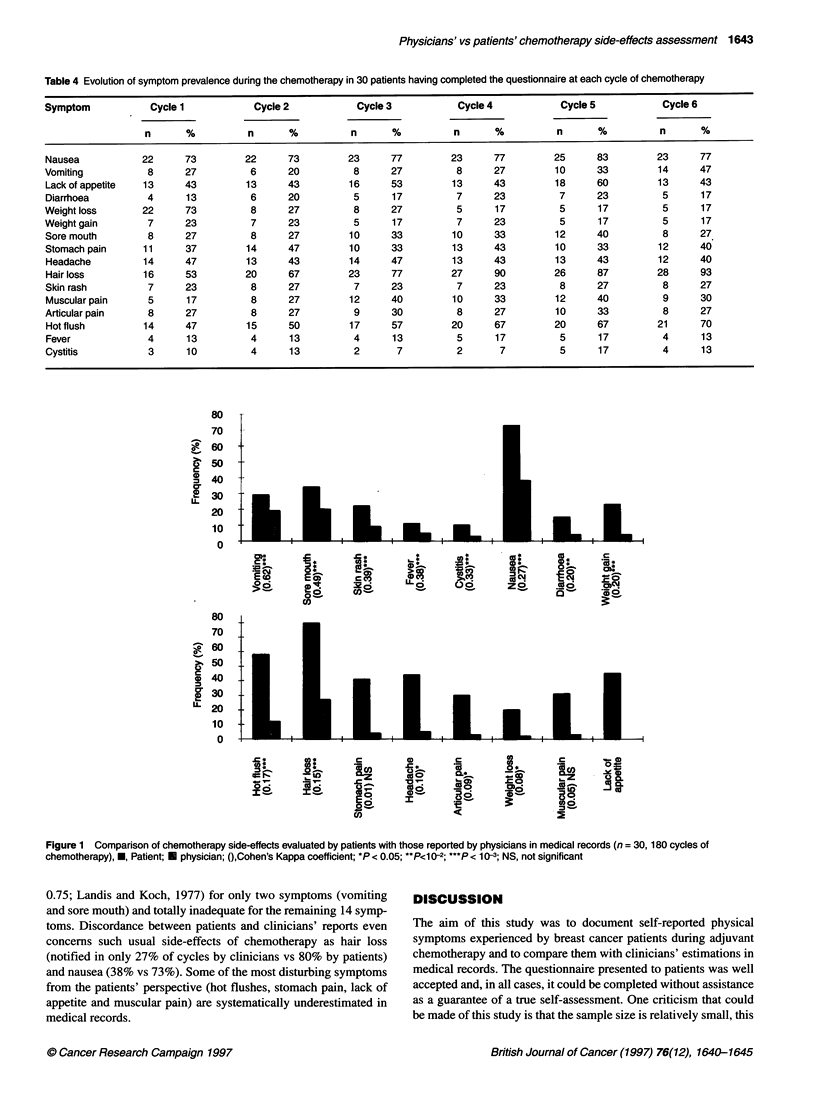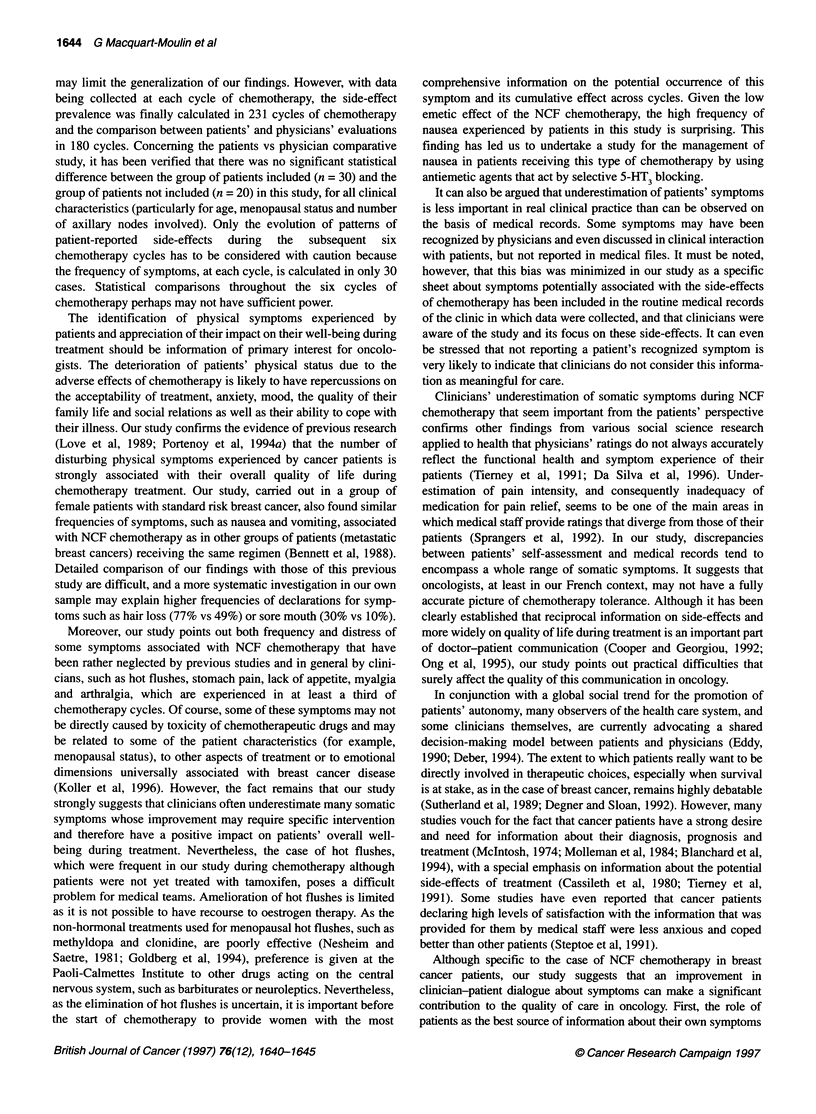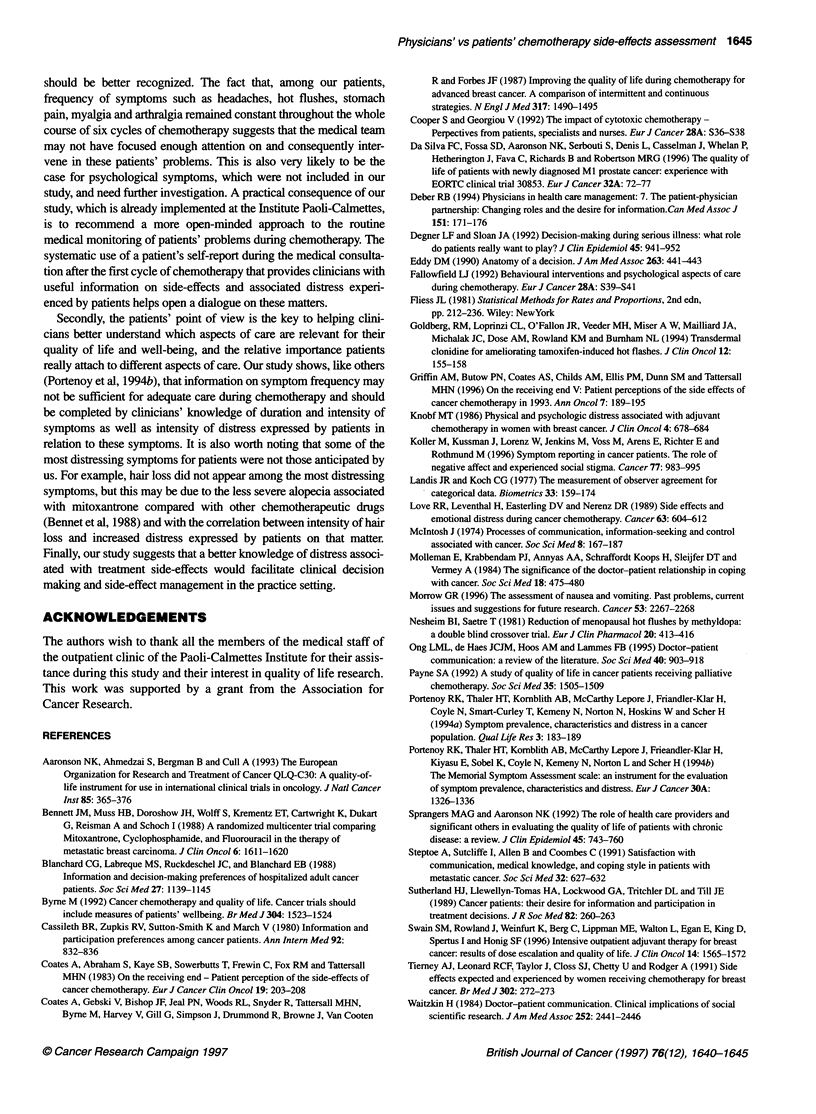# Discordance between physicians' estimations and breast cancer patients' self-assessment of side-effects of chemotherapy: an issue for quality of care.

**DOI:** 10.1038/bjc.1997.610

**Published:** 1997

**Authors:** G. Macquart-Moulin, P. Viens, M. L. Bouscary, D. Genre, M. Resbeut, G. Gravis, J. Camerlo, D. Maraninchi, J. P. Moatti

**Affiliations:** INSERM, Research unit no. 379 Epidemiology and social sciences applied to medical innovation, Marseilles, France.

## Abstract

Because side-effects of chemotherapy may be more diverse and patients' reactions more individualistic than tends to be acknowledged by clinicians, a survey was carried out among 50 breast cancer outpatients to document self-reported physical symptoms experienced during NCF (mitoxantrone + cyclophosphamide + 5-fluorouracil) adjuvant chemotherapy and to compare them with the clinicians' estimation in medical records. The questionnaire evaluated the prevalence, duration/severity and distress level of 17 symptoms. Symptom prevalence, assessed in 231 cycles, was high even for symptoms that do not usually focus clinicians' attention. Of these, hot flushes, stomach pain and muscular and articular pains lasted 1 week or more for nearly half of the cycles. Hot flushes, vomiting and stomach pain were the most distressing symptoms. The mean number of symptoms per cycle is significantly correlated with the global quality-of-life score. Concordance between patients' self-assessment and clinical reports, measured in 180 cycles, is moderately correct for vomiting and sore mouth and inadequate for the remaining symptoms even for hair loss (notified in 27% of cycles by clinicians vs 80% by patients) and nausea (38% vs 73%). A better understanding by physicians of cancer patients' problems is necessary to improve quality of care.


British Journal of Cancer (1997) 76(12), 1640–1645
© 1997 Cancer Research Campaign

### Discordance between physicians' estimations and breast cancer patients' self-assessment of side-effects of chemotherapy: an issue for quality of care

G Macquart-Moulin[1], P Viens[1,2], M-L Bouscary[1], D Genre[1,2], M Resbeut[2], G Gravis[2], J Camerlo[2], D Maraninchi[2,3] and J-P Moatti[1,3]

[1]INSERM, Research unit no. 379 'Epidemiology and social sciences applied to medical innovation', Marseilles; [2]Institut Paoli-Calmettes, Regional Center for Cancer Research and Treatment, Marseilles; [3]University of the Mediterranean (Aix-Marseilles II), France

**Summary** Because side-effects of chemotherapy may be more diverse and patients' reactions more individualistic than tends to be acknowledged by clinicians, a survey was carried out among 50 breast cancer outpatients to document self-reported physical symptoms experienced during NCF (mitoxantrone + cyclophosphamide + 5-fluorouracil) adjuvant chemotherapy and to compare them with the clinicians' estimation in medical records. The questionnaire evaluated the prevalence, duration/severity and distress level of 17 symptoms. Symptom prevalence, assessed in 231 cycles, was high even for symptoms that do not usually focus clinicians' attention. Of these, hot flushes, stomach pain and muscular and articular pains lasted 1 week or more for nearly half of the cycles. Hot flushes, vomiting and stomach pain were the most distressing symptoms. The mean number of symptoms per cycle is significantly correlated with the global quality-of-life score. Concordance between patients' self-assessment and clinical reports, measured in 180 cycles, is moderately correct for vomiting and sore mouth and inadequate for the remaining symptoms even for hair loss (notified in 27% of cycles by clinicians vs 80% by patients) and nausea (38% vs 73%). A better understanding by physicians of cancer patients' problems is necessary to improve quality of care.

**Keywords:** breast cancer; chemotherapy; physical symptoms; quality of life; physician–patient communication

It is well established that somatic symptoms condition cancer patients' acceptability of treatments and that side-effects of chemotherapy may create barriers to patients' acceptance and compliance with effective adjuvant therapies before and during treatment (Love et al, 1989; Cooper and Georgiou, 1992; Fallowfield, 1992). Oncologists' attention has, however, been mainly focused on toxicity of chemotherapy and on some of its most noticeable associated side-effects (hair loss, nausea and vomiting) (Coates et al, 1983; Griffin et al, 1996; Morrow, 1996). In recent years, there have been more systematic attempts to measure the impact of side-effects on patients' well-being and quality of life during chemotherapy treatments by taking into account patients' subjective point of view (Coates et al, 1983, 1987; Knobf, 1986; Byrne, 1992; Payne, 1992; Griffin et al, 1996; Swain et al, 1996). However, it is obvious that such approaches have not yet been fully integrated in day-to-day clinical practice in oncology (Waitzkin, 1984; Sutherland et al, 1989).

Because side-effects of chemotherapy may be more diverse and reactions of patients more variable and individualistic than tends to be acknowledged by clinicians, we have carried out a study among standard risk breast cancer patients to compare their own self-assessment of physical symptoms and associated distress they experience during adjuvant chemotherapy with the estimation by clinicians as it appears in medical records.

*Received 20 November 1996*
*Revised 13 March 1997*
*Accepted 4 June 1997*

*Correspondence to:* G Macquart-Moulin, INSERM U 379, Institut Paoli-Calmettes, 232, Boulevard de Sainte-Marguerite, 13273 Marseille Cedex 9, France

### PATIENTS AND METHODS

### Patient selection

The study was carried out in the outpatient clinic of the Paoli-Calmettes Institute (Regional Center for Cancer Care and Research of Marseilles in south-eastern France) between July 1994 and July 1995. All breast cancer patients with no metastases and less than nine axillary nodes involved, receiving an adjuvant NCF polychemotherapy, comprising Mitoxantrone (12 mg $m^{-2}$), cyclophosphamide (500 mg $m^{-2}$), and 5-fluorouracil (500 mg $m^{-2}$), i.v. administered in six cycles of 21 days, and concomitant radiotherapy were included in the study. Tamoxifen (20 mg daily) was given for 3 years to women with confirmed menopausal status at diagnosis and with positive hormonal receptors. It was started only at the end of the chemotherapy and radiotherapy sequence.

### Patients' self-assessment

Before starting a new cycle of chemotherapy, each patient was asked to complete a written self-administered questionnaire about the side-effects she had experienced during the previous cycle and her global quality of life during this period. The last questionnaire, assessing the side-effects effects related to the sixth cycle of chemotherapy, was completed by the patient at home and returned to the medical team during a follow-up consultation or sent by mail with a stamped-addressed envelope, Each questionnaire took approximately 10 min to complete.

The questionnaire included a list of 17 symptoms commonly associated with chemotherapy for breast cancer: nausea, vomiting, lack of appetite, diarrhoea, weight loss and gain, sore mouth, stomach pain, headache, hair loss, skin rash, articular and muscular pain, cystitis, menstruation problem, hot flush and fever.

For each symptom, patients were asked three times: (a) if they had experienced this symptom at least once during the previous 3 weeks (yes or no); (b) what was the duration of such a symptom (using a four-point scale: less than 2 days, 3–6 days, 1 or 2 weeks, all the time); and (c) to what extent the symptom had been disturbing for the patient (using a four-point Likert scale: not at all, a little bit, quite a bit, very much). Because duration was not relevant for three symptoms (hair and weight loss/gain), severity was evaluated instead.

Global ratings of physical status and quality of life during the previous 3 weeks were also obtained using similar items to those included in the EORTC QLQ-C30 questionnaire (items 29 and 30) (Aaronson et al, 1993). A score of 1 was assigned to a 'very poor' physical status or global quality of life and 7 to an 'excellent' physical status or global quality of life. At the end of the questionnaire, a comment section was added and patients were encouraged to report any other physical problems experienced during the chemotherapy course, as well as their duration/severity and the distress that was associated with it.

### Physicians' assessment

Physicians' evaluations of the same 17 symptoms, estimated by them for each cycle of chemotherapy, were directly collected from medical records. A standardized sheet about toxicity of treatment, containing the same list of symptoms as in the patients' questionnaire, had been introduced 6 months previously in the routine medical record of the clinic. At the time of the consultation, carried out before the start of each cycle of chemotherapy, clinicians routinely asked the women about the side-effects they had experienced during the previous cycle. The standard sheet is used to guide them in this task and to make reporting of symptoms easier. Symptoms are assessed in terms of presence or absence.

### Data analysis

A questionnaire was excluded from analysis if the answers to more than 10% of items were missing, duration/severity and distress associated with each symptom being considered as separate items for this calculation. The missing values were treated as follows: if, for example, in the questionnaire completed by a patient at the third cycle of chemotherapy, duration of nausea was missing, we assigned the median value of patients' answers to this item for the same third cycle of treatment. A global quality-of-life score (QOL score) was calculated as the mean of the scores on the two scales corresponding to global health and global quality of life. This score was linearly transformed to a 0–100 score, with a higher score representing a higher level of global quality of life (Aaronson et al, 1993).

Spearman coefficients were calculated to investigate correlations between answers about duration/severity of each symptom and answers about the distress associated with each of them, as well as correlations between mean number of declared symptoms during each cycle of chemotherapy and global QOL score. Cochran and Friedman non-parametric tests were used to test for significant changes over time among the subgroup of patients who completed a questionnaire at each of the six cycles of chemotherapy ($n$ = 30). To compare patients' declarations about prevalence of symptoms with those reported by the physicians,

**Table 1** Clinical characteristics of the consenting patients ($n$ = 50)

| Characteristic | $n$ | % |
|---|---|---|
| Histological type | | |
| Ductal adenocarcinoma | 34 | 68 |
| Lobular adenocarcinoma | 3 | 6 |
| Other histological type | 13 | 26 |
| AJCC stage | | |
| I | 18 | 36 |
| II | 25 | 50 |
| III | 3 | 6 |
| Not applicable* | 4 | 8 |
| SBR grading | | |
| I | 7 | 14 |
| II | 23 | 46 |
| III | 16 | 32 |
| Not done | 4 | 8 |
| Axillary nodes | | |
| N- | 14 | 28 |
| 1–3 | 28 | 56 |
| >3 | 8 | 16 |
| Hormonal receptors | | |
| Positive | 23 | 46 |
| Negative | 20 | 40 |
| Unknown | 7 | 14 |
| Menopausal status | | |
| Menopausal | 24 | 48 |
| Non-menopausal | 26 | 52 |

*These four patients presented a local relapse but they had not received earlier chemotherapy.

Cohen's Kappa coefficient was used as the measure of agreement between the two types of evaluation (Fliess, 1981).

### RESULTS

Fifty-two patients, aged between 32 and 70 years (median = 51), were asked to participate in the study, starting at different cycles of their treatment. Only two patients refused to participate (participation rate 96%). A subgroup of 33 patients were asked to participate for the whole course of their treatment, that is from the first to the sixth cycle of chemotherapy. Three patients in this group did not complete one of the six questionnaires.

After exclusion of one questionnaire with more than 10% of items not answered, a total of 231 questionnaires were available for statistical analysis 40, 43, 40, 38, 38 and 32 from the first to the sixth cycle of chemotherapy respectively. The average rate of missing values was 2.3% for the items of the duration/severity dimension and 6% for the items on the distress dimension. It must be noted that, for the calculations of missing values as well as for the further analyses, the item 'menstruation problems' was discarded because of the unsuitable formulation of the corresponding question.

All the patients were able to complete the questionnaire without assistance. Most of them had no problem in understanding questions, except the one relating to the above-mentioned symptom. The clinical characteristics of the consenting patients ($n$ = 50) are displayed in Table 1.

Table 2 summarizes the patients' declared frequency of symptoms, their duration/severity and the degree of distress that patients

© Cancer Research Campaign 1997

**Table 2** Prevalence and characteristics of symptoms, when present, evaluated by the study patients in 231 chemotherapy cycles

| | Frequency | | Duration/severity[a] | | | Distress[a] | | |
|---|---|---|---|---|---|---|---|---|
| Symptom | n | % | 1–2 days[b] % | 3–6 days[c] % | ≥ 7 days[d] % | Not at all % | A little bit % | Quite a bit/very much % |
| Hair loss | 178 | 77 | 50 | 32 | 18 | 14 | 36 | 50 |
| Nausea | 176 | 76 | 25 | 54 | 21 | 2 | 41 | 57 |
| Hot flush | 134 | 58 | 10 | 18 | 72 | 1 | 22 | 77 |
| Lack of appetite | 109 | 47 | 23 | 39 | 38 | 23 | 25 | 52 |
| Headache | 102 | 44 | 46 | 40 | 14 | 4 | 55 | 41 |
| Stomach pain | 88 | 38 | 15 | 39 | 46 | 1 | 28 | 71 |
| Sore mouth | 69 | 30 | 23 | 38 | 39 | 4 | 46 | 50 |
| Muscular pain | 69 | 30 | 26 | 25 | 49 | 3 | 46 | 51 |
| Vomiting | 67 | 29 | 46 | 51 | 3 | 2 | 25 | 73 |
| Articular pain | 65 | 28 | 29 | 25 | 46 | 7 | 48 | 45 |
| Skin rash | 55 | 24 | 38 | 27 | 35 | 7 | 47 | 46 |
| Weight loss | 51 | 22 | 44 | 32 | 24 | 53 | 29 | 18 |
| Weight gain | 44 | 19 | 45 | 33 | 22 | 26 | 39 | 35 |
| Diarrhoea | 39 | 17 | 58 | 33 | 9 | 24 | 43 | 33 |
| Fever | 30 | 13 | 55 | 32 | 13 | 14 | 39 | 47 |
| Cystitis | 23 | 10 | 48 | 17 | 35 | 4 | 54 | 42 |

[a]When the symptom is present. The number of respondents is shown in the first column; it may vary slightly because of missing data.
[b]Hair loss, a little bit; weight loss/gain, 1 kg; [c]Hair loss, quite a bit, weight loss/gain, 2 kg; [d]Hair loss, very much/completely; weight loss/gain, ≥3 kg.

**Table 3** Correlation between the mean number of symptoms and QOL score at each cycle of chemotherapy

| | Cycle 1 ($n = 40$) | Cycle 2 ($n = 43$) | Cycle 3 ($n = 40$) | Cycle 4 ($n = 38$) | Cycle 5 ($n = 38$) | Cycle 6 ($n = 32$) |
|---|---|---|---|---|---|---|
| Mean number of symptoms | 5.5 ± 3.1 | 5.7 ± 2.9 | 6.0 ± 3.1 | 6.2 ± 2.7 | 6.8 ± 2.7 | 6.3 ± 2.4 |
| Mean QOL score | 59.7 ± 18.8 | 57.5 ± 19.9 | 51.9 ± 21.7 | 51.9 ± 16.6 | 48.5 ± 15.7 | 46.9 ± 15.9 |
| Correlation coefficient ($r$) | –0.37* | –0.44 | –0.60** | –0.49 | –0.47 | –0.45 |

*$P$<0.05; **$P$<0.001; $P$<0.01 for all other statistics.

associated with them during the 231 cycles of chemotherapy for which we have valid information. Not surprisingly, frequency is high even for symptoms such as hot flushes, lack of appetite, headaches, stomach pain and muscular and articular pains, which do not usually attract a lot of clinical attention. Some of these frequent symptoms (hot flushes, stomach, muscular and articular pains) tended to last 1 week or more for nearly half of the cycles. When present, sore mouth, lack of appetite, skin rashes and cystits also last at least 1 week for more than a third of cycles. Although the most frequent, hair loss is considered as severe for only 18% of cycles. Hot flushes, vomiting and stomach pain are the most disturbing symptoms for the patients. In addition, in the open section, tiredness, change in taste and conjunctivitis were symptoms spontaneously mentioned by patients in more than 10% of the questionnaires.

For each symptom, mean scores were calculated, on all the cycles of chemotherapy, for the answers relating to duration/severity and the answers about degree of associated distress. Pairwise interdimension correlations were calculated between these two scores and revealed statistically significant relationships ($P < 0.05$) between duration/severity of a symptom and its impact on patient's distress in the cases of lack of appetite ($r = 0.92$), diarrhoea ($r = 0.69$), hot flush ($r = 0.55$), headache ($r = 0.54$), weight gain ($r = 0.54$), hair loss ($r = 0.51$) and articular pain ($r = 0.51$). It must be noted that no statistically significant relation was found between duration of episodes of vomiting or nausea and patients' associated distress, suggesting that even limited episodes may be sufficient to disturb patients.

Table 3 shows that the mean number of symptoms declared per cycle by patients is significantly correlated with their global quality-of-life score during this cycle: the more patients experience physical symptoms during a cycle of chemotherapy, the lower their QOL score during this period. Moreover, global quality of life, already moderate during the first cycle of chemotherapy, regularly decreases over time, whereas the number of physical symptoms increases.

In the subgroup of 30 patients who completed a questionnaire for each of their six chemotherapy cycles (Table 4), the declared frequency of symptoms tended to remain constant across cycles, with two exceptions: hot flushes, which were only mentioned by 47% of patients at first cycle but 70% at the last one, and hair loss, which increased from 53% to 93% of patients between cycle 1 and cycle 6. Nevertheless, only frequency of hair loss demonstrates a statistically significant change over time ($P < 0.001$). The mean score for severity of hair loss also significantly increases (from 1.1 ± 0.3 at cycle 1 to 1.8 ± 0.7 at cycle 6, $P < 0.05$) as well as the mean score for associated distress (1.9 ± 0.8 vs 2.7 ± 0.9, $P < 0.05$).

Figure 1 compares the frequency of declaration of each symptom, during the 180 cycles of chemotherapy of these 30 patients, to those noted by clinicians in their medical records. Concordance between patient's self-assessment and clinical reports is moderately correct (Kappa coefficient between 0.45 and

 © Cancer Research Campaign 1997

**Table 4** Evolution of symptom prevalence during the chemotherapy in 30 patients having completed the questionnaire at each cycle of chemotherapy

| Symptom | Cycle 1 | | Cycle 2 | | Cycle 3 | | Cycle 4 | | Cycle 5 | | Cycle 6 | |
|---|---|---|---|---|---|---|---|---|---|---|---|---|
| | n | % | n | % | n | % | n | % | n | % | n | % |
| Nausea | 22 | 73 | 22 | 73 | 23 | 77 | 23 | 77 | 25 | 83 | 23 | 77 |
| Vomiting | 8 | 27 | 6 | 20 | 8 | 27 | 8 | 27 | 10 | 33 | 14 | 47 |
| Lack of appetite | 13 | 43 | 13 | 43 | 16 | 53 | 13 | 43 | 18 | 60 | 13 | 43 |
| Diarrhoea | 4 | 13 | 6 | 20 | 5 | 17 | 7 | 23 | 7 | 23 | 5 | 17 |
| Weight loss | 22 | 73 | 8 | 27 | 8 | 27 | 5 | 17 | 5 | 17 | 5 | 17 |
| Weight gain | 7 | 23 | 7 | 23 | 5 | 17 | 7 | 23 | 5 | 17 | 5 | 17 |
| Sore mouth | 8 | 27 | 8 | 27 | 10 | 33 | 10 | 33 | 12 | 40 | 8 | 27 |
| Stomach pain | 11 | 37 | 14 | 47 | 10 | 33 | 13 | 43 | 10 | 33 | 12 | 40 |
| Headache | 14 | 47 | 13 | 43 | 14 | 47 | 13 | 43 | 13 | 43 | 12 | 40 |
| Hair loss | 16 | 53 | 20 | 67 | 23 | 77 | 27 | 90 | 26 | 87 | 28 | 93 |
| Skin rash | 7 | 23 | 8 | 27 | 7 | 23 | 7 | 23 | 8 | 27 | 8 | 27 |
| Muscular pain | 5 | 17 | 8 | 27 | 12 | 40 | 10 | 33 | 12 | 40 | 9 | 30 |
| Articular pain | 8 | 27 | 8 | 27 | 9 | 30 | 8 | 27 | 10 | 33 | 8 | 27 |
| Hot flush | 14 | 47 | 15 | 50 | 17 | 57 | 20 | 67 | 20 | 67 | 21 | 70 |
| Fever | 4 | 13 | 4 | 13 | 4 | 13 | 5 | 17 | 5 | 17 | 4 | 13 |
| Cystitis | 3 | 10 | 4 | 13 | 2 | 7 | 2 | 7 | 5 | 17 | 4 | 13 |

**Figure 1** Comparison of chemotherapy side-effects evaluated by patients with those reported by physicians in medical records ($n$ = 30, 180 cycles of chemotherapy), ■, Patient; ■ physician; (),Cohen's Kappa coefficient; $*P < 0.05$; $**P<10^{-2}$; $***P < 10^{-3}$; NS, not significant

0.75; Landis and Koch, 1977) for only two symptoms (vomiting and sore mouth) and totally inadequate for the remaining 14 symptoms. Discordance between patients and clinicians' reports even concerns such usual side-effects of chemotherapy as hair loss (notified in only 27% of cycles by clinicians vs 80% by patients) and nausea (38% vs 73%). Some of the most disturbing symptoms from the patients' perspective (hot flushes, stomach pain, lack of appetite and muscular pain) are systematically underestimated in medical records.

### DISCUSSION

The aim of this study was to document self-reported physical symptoms experienced by breast cancer patients during adjuvant chemotherapy and to compare them with clinicians' estimations in medical records. The questionnaire presented to patients was well accepted and, in all cases, it could be completed without assistance as a guarantee of a true self-assessment. One criticism that could be made of this study is that the sample size is relatively small, this may limit the generalization of our findings. However, with data being collected at each cycle of chemotherapy, the side-effect prevalence was finally calculated in 231 cycles of chemotherapy and the comparison between patients' and physicians' evaluations in 180 cycles. Concerning the patients vs physician comparative study, it has been verified that there was no significant statistical difference between the group of patients included ($n = 30$) and the group of patients not included ($n = 20$) in this study, for all clinical characteristics (particularly for age, menopausal status and number of axillary nodes involved). Only the evolution of patterns of patient-reported side-effects during the subsequent six chemotherapy cycles has to be considered with caution because the frequency of symptoms, at each cycle, is calculated in only 30 cases. Statistical comparisons throughout the six cycles of chemotherapy perhaps may not have sufficient power.

The identification of physical symptoms experienced by patients and appreciation of their impact on their well-being during treatment should be information of primary interest for oncologists. The deterioration of patients' physical status due to the adverse effects of chemotherapy is likely to have repercussions on the acceptability of treatment, anxiety, mood, the quality of their family life and social relations as well as their ability to cope with their illness. Our study confirms the evidence of previous research (Love et al, 1989; Portenoy et al, 1994*a*) that the number of disturbing physical symptoms experienced by cancer patients is strongly associated with their overall quality of life during chemotherapy treatment. Our study, carried out in a group of female patients with standard risk breast cancer, also found similar frequencies of symptoms, such as nausea and vomiting, associated with NCF chemotherapy as in other groups of patients (metastatic breast cancers) receiving the same regimen (Bennett et al, 1988). Detailed comparison of our findings with those of this previous study are difficult, and a more systematic investigation in our own sample may explain higher frequencies of declarations for symptoms such as hair loss (77% vs 49%) or sore mouth (30% vs 10%).

Moreover, our study points out both frequency and distress of some symptoms associated with NCF chemotherapy that have been rather neglected by previous studies and in general by clinicians, such as hot flushes, stomach pain, lack of appetite, myalgia and arthralgia, which are experienced in at least a third of chemotherapy cycles. Of course, some of these symptoms may not be directly caused by toxicity of chemotherapeutic drugs and may be related to some of the patient characteristics (for example, menopausal status), to other aspects of treatment or to emotional dimensions universally associated with breast cancer disease (Koller et al, 1996). However, the fact remains that our study strongly suggests that clinicians often underestimate many somatic symptoms whose improvement may require specific intervention and therefore have a positive impact on patients' overall well-being during treatment. Nevertheless, the case of hot flushes, which were frequent in our study during chemotherapy although patients were not yet treated with tamoxifen, poses a difficult problem for medical teams. Amelioration of hot flushes is limited as it is not possible to have recourse to oestrogen therapy. As the non-hormonal treatments used for menopausal hot flushes, such as methyldopa and clonidine, are poorly effective (Nesheim and Saetre, 1981; Goldberg et al, 1994), preference is given at the Paoli-Calmettes Institute to other drugs acting on the central nervous system, such as barbiturates or neuroleptics. Nevertheless, as the elimination of hot flushes is uncertain, it is important before the start of chemotherapy to provide women with the most comprehensive information on the potential occurrence of this symptom and its cumulative effect across cycles. Given the low emetic effect of the NCF chemotherapy, the high frequency of nausea experienced by patients in this study is surprising. This finding has led us to undertake a study for the management of nausea in patients receiving this type of chemotherapy by using antiemetic agents that act by selective $5\text{-HT}_3$ blocking.

It can also be argued that underestimation of patients' symptoms is less important in real clinical practice than can be observed on the basis of medical records. Some symptoms may have been recognized by physicians and even discussed in clinical interaction with patients, but not reported in medical files. It must be noted, however, that this bias was minimized in our study as a specific sheet about symptoms potentially associated with the side-effects of chemotherapy has been included in the routine medical records of the clinic in which data were collected, and that clinicians were aware of the study and its focus on these side-effects. It can even be stressed that not reporting a patient's recognized symptom is very likely to indicate that clinicians do not consider this information as meaningful for care.

Clinicians' underestimation of somatic symptoms during NCF chemotherapy that seem important from the patients' perspective confirms other findings from various social science research applied to health that physicians' ratings do not always accurately reflect the functional health and symptom experience of their patients (Tierney et al, 1991; Da Silva et al, 1996). Underestimation of pain intensity, and consequently inadequacy of medication for pain relief, seems to be one of the main areas in which medical staff provide ratings that diverge from those of their patients (Sprangers et al, 1992). In our study, discrepancies between patients' self-assessment and medical records tend to encompass a whole range of somatic symptoms. It suggests that oncologists, at least in our French context, may not have a fully accurate picture of chemotherapy tolerance. Although it has been clearly established that reciprocal information on side-effects and more widely on quality of life during treatment is an important part of doctor–patient communication (Cooper and Georgiou, 1992; Ong et al, 1995), our study points out practical difficulties that surely affect the quality of this communication in oncology.

In conjunction with a global social trend for the promotion of patients' autonomy, many observers of the health care system, and some clinicians themselves, are currently advocating a shared decision-making model between patients and physicians (Eddy, 1990; Deber, 1994). The extent to which patients really want to be directly involved in therapeutic choices, especially when survival is at stake, as in the case of breast cancer, remains highly debatable (Sutherland et al, 1989; Degner and Sloan, 1992). However, many studies vouch for the fact that cancer patients have a strong desire and need for information about their diagnosis, prognosis and treatment (McIntosh, 1974; Molleman et al, 1984; Blanchard et al, 1994), with a special emphasis on information about the potential side-effects of treatment (Cassileth et al, 1980; Tierney et al, 1991). Some studies have even reported that cancer patients declaring high levels of satisfaction with the information that was provided for them by medical staff were less anxious and coped better than other patients (Steptoe et al, 1991).

Although specific to the case of NCF chemotherapy in breast cancer patients, our study suggests that an improvement in clinician–patient dialogue about symptoms can make a significant contribution to the quality of care in oncology. First, the role of patients as the best source of information about their own symptoms

© Cancer Research Campaign 1997

should be better recognized. The fact that, among our patients, frequency of symptoms such as headaches, hot flushes, stomach pain, myalgia and arthralgia remained constant throughout the whole course of six cycles of chemotherapy suggests that the medical team may not have focused enough attention on and consequently intervene in these patients' problems. This is also very likely to be the case for psychological symptoms, which were not included in our study, and need further investigation. A practical consequence of our study, which is already implemented at the Institute Paoli-Calmettes, is to recommend a more open-minded approach to the routine medical monitoring of patients' problems during chemotherapy. The systematic use of a patient's self-report during the medical consultation after the first cycle of chemotherapy that provides clinicians with useful information on side-effects and associated distress experienced by patients helps open a dialogue on these matters.

Secondly, the patients' point of view is the key to helping clinicians better understand which aspects of care are relevant for their quality of life and well-being, and the relative importance patients really attach to different aspects of care. Our study shows, like others (Portenoy et al, 1994*b*), that information on symptom frequency may not be sufficient for adequate care during chemotherapy and should be completed by clinicians' knowledge of duration and intensity of symptoms as well as intensity of distress expressed by patients in relation to these symptoms. It is also worth noting that some of the most distressing symptoms for patients were not those anticipated by us. For example, hair loss did not appear among the most distressing symptoms, but this may be due to the less severe alopecia associated with mitoxantrone compared with other chemotherapeutic drugs (Bennet et al, 1988) and with the correlation between intensity of hair loss and increased distress expressed by patients on that matter. Finally, our study suggests that a better knowledge of distress associated with treatment side-effects would facilitate clinical decision making and side-effect management in the practice setting.

### ACKNOWLEDGEMENTS

The authors wish to thank all the members of the medical staff of the outpatient clinic of the Paoli-Calmettes Institute for their assistance during this study and their interest in quality of life research. This work was supported by a grant from the Association for Cancer Research.

### REFERENCES

Aaronson NK, Ahmedzai S, Bergman B and Cull A (1993) The European Organization for Research and Treatment of Cancer QLQ-C30: A quality-of-life instrument for use in international clinical trials in oncology. *J Natl Cancer Inst* **85**: 365–376

Bennett JM, Muss HB, Doroshow JH, Wolff S, Krementz ET, Cartwright K, Dukart G, Reisman A and Schoch I (1988) A randomized multicenter trial comparing Mitoxantrone, Cyclophosphamide, and Fluorouracil in the therapy of metastatic breast carcinoma. *J Clin Oncol* **6**: 1611–1620

Blanchard CG, Labreque MS, Ruckdeschel JC, and Blanchard EB (1988) Information and decision-making preferences of hospitalized adult cancer patients. *Soc Sci Med* **27**: 1139–1145

Byrne M (1992) Cancer chemotherapy and quality of life. Cancer trials should include measures of patients' wellbeing. *Br Med J* **304**: 1523–1524

Cassileth BR, Zupkis RV, Sutton-Smith K and March V (1980) Information and participation preferences among cancer patients. *Ann Intern Med* **92**: 832–836

Coates A, Abraham S, Kaye SB, Sowerbutts T, Frewin C, Fox RM and Tattersall MHN (1983) On the receiving end – Patient perception of the side-effects of cancer chemotherapy. *Eur J Cancer Clin Oncol* **19**: 203–208

Coates A, Gebski V, Bishop JF, Jeal PN, Woods RL, Snyder R, Tattersall MHN, Byrne M, Harvey V, Gill G, Simpson J, Drummond R, Browne J, Van Cooten R and Forbes JF (1987) Improving the quality of life during chemotherapy for advanced breast cancer. A comparison of intermittent and continuous strategies. *N Engl J Med* **317**: 1490–1495

Cooper S and Georgiou V (1992) The impact of cytotoxic chemotherapy – Perpectives from patients, specialists and nurses. *Eur J Cancer* **28A**: S36–S38

Da Silva FC, Fossa SD, Aaronson NK, Serbouti S, Denis L, Casselman J, Whelan P, Hetherington J, Fava C, Richards B and Robertson MRG (1996) The quality of life of patients with newly diagnosed M1 prostate cancer: experience with EORTC clinical trial 30853. *Eur J Cancer* **32A**: 72–77

Deber RB (1994) Physicians in health care management: 7. The patient-physician partnership: Changing roles and the desire for information. *Can Med Assoc J* **151**: 171–176

Degner LF and Sloan JA (1992) Decision-making during serious illness: what role do patients really want to play? *J Clin Epidemiol* **45**: 941–952

Eddy DM (1990) Anatomy of a decision. *J Am Med Assoc* **263**: 441–443

Fallowfield LJ (1992) Behavioural interventions and psychological aspects of care during chemotherapy. *Eur J Cancer* **28A**: S39–S41

Fliess JL (1981) *Statistical Methods for Rates and Proportions*, 2nd edn, pp. 212–236. Wiley: NewYork

Goldberg, RM, Loprinzi CL, O'Fallon JR, Veeder MH, Miser A W, Mailliard JA, Michalak JC, Dose AM, Rowland KM and Burnham NL (1994) Transdermal clonidine for ameliorating tamoxifen-induced hot flashes. *J Clin Oncol* **12**: 155–158

Griffin AM, Butow PN, Coates AS, Childs AM, Ellis PM, Dunn SM and Tattersall MHN (1996) On the receiving end V: Patient perceptions of the side effects of cancer chemotherapy in 1993. *Ann Oncol* **7**: 189–195

Knobf MT (1986) Physical and psychologic distress associated with adjuvant chemotherapy in women with breast cancer. *J Clin Oncol* **4**: 678–684

Koller M, Kussman J, Lorenz W, Jenkins M, Voss M, Arens E, Richter E and Rothmund M (1996) Symptom reporting in cancer patients. The role of negative affect and experienced social stigma. *Cancer* **77**: 983–995

Landis JR and Koch CG (1977) The measurement of observer agreement for categorical data. *Biometrics* **33**: 159–174

Love RR, Leventhal H, Easterling DV and Nerenz DR (1989) Side effects and emotional distress during cancer chemotherapy. *Cancer* **63**: 604–612

McIntosh J (1974) Processes of communication, information-seeking and control associated with cancer. *Soc Sci Med* **8**: 167–187

Molleman E, Krabbendam PJ, Annyas AA, Schraffordt Koops H, Sleijfer DT and Vermey A (1984) The significance of the doctor–patient relationship in coping with cancer. *Soc Sci Med* **18**: 475–480

Morrow GR (1996) The assessment of nausea and vomiting. Past problems, current issues and suggestions for future research. *Cancer* **53**: 2267–2268

Nesheim BI, Saetre T (1981) Reduction of menopausal hot flushes by methyldopa: a double blind crossover trial. *Eur J Clin Pharmacol* **20**: 413–416

Ong LML, de Haes JCJM, Hoos AM and Lammes FB (1995) Doctor–patient communication: a review of the literature. *Soc Sci Med* **40**: 903–918

Payne SA (1992) A study of quality of life in cancer patients receiving palliative chemotherapy. *Soc Sci Med* **35**: 1505–1509

Portenoy RK, Thaler HT, Kornblith AB, McCarthy Lepore J, Friandler-Klar H, Coyle N, Smart-Curley T, Kemeny N, Norton N, Hoskins W and Scher H (1994*a*) Symptom prevalence, characteristics and distress in a cancer population. *Qual Life Res* **3**: 183–189

Portenoy RK, Thaler HT, Kornblith AB, McCarthy Lepore J, Frieandler-Klar H, Kiyasu E, Sobel K, Coyle N, Kemeny N, Norton L and Scher H (1994*b*) The Memorial Symptom Assessment scale: an instrument for the evaluation of symptom prevalence, characteristics and distress. *Eur J Cancer* **30A**: 1326–1336

Sprangers MAG and Aaronson NK (1992) The role of health care providers and significant others in evaluating the quality of life of patients with chronic disease: a review. *J Clin Epidemiol* **45**: 743–760

Steptoe A, Sutcliffe I, Allen B and Coombes C (1991) Satisfaction with communication, medical knowledge, and coping style in patients with metastatic cancer. *Soc Sci Med* **32**: 627–632

Sutherland HJ, Llewellyn-Tomas HA, Lockwood GA, Tritchler DL and Till JE (1989) Cancer patients: their desire for information and participation in treatment decisions. *J R Soc Med* **82**: 260–263

Swain SM, Rowland J, Weinfurt K, Berg C, Lippman ME, Walton L, Egan E, King D, Spertus I and Honig SF (1996) Intensive outpatient adjuvant therapy for breast cancer: results of dose escalation and quality of life. *J Clin Oncol* **14**: 1565–1572

Tierney AJ, Leonard RCF, Taylor J, Closs SJ, Chetty U and Rodger A (1991) Side effects expected and experienced by women receiving chemotherapy for breast cancer. *Br Med J* **302**: 272–273

Waitzkin H (1984) Doctor–patient communication. Clinical implications of social scientific research. *J Am Med Assoc* **252**: 2441–2446

© Cancer Research Campaign 1997